# Hypertrophic Cardiomyopathy Diagnosis and Treatment in High- and Low-Income Countries: A Narrative Review

**DOI:** 10.7759/cureus.46330

**Published:** 2023-10-01

**Authors:** Ernesto Calderon Martinez, Nancy Y Ortiz-Garcia, Domenica A Herrera Hernandez, David Arriaga Escamilla, Diana L Diaz Mendoza, Diana Othon Martinez, Luz M Ramirez, Jonathan Reyes-Rivera, Jinal Choudhari, George Michel

**Affiliations:** 1 Biomedical Informatics, Universidad Nacional Autónoma de México, Mexico City, MEX; 2 Integrative Medicine, Universidad Juárez del Estado de Durango, Durango, MEX; 3 Internal Medicine, Pontifical Catholic University of Ecuador, Quito, ECU; 4 Internal Medicine, Universidad Justo Sierra, Mexico City, MEX; 5 Internal Medicine, Universidad Nacional Autonoma de Mexico, Mexico City, MEX; 6 Internal Medicine, University of Texas Rio Grande Valley, Edinburg, USA; 7 Pulmonology and Critical Care, Benemerita Universidad Autonoma de Puebla, Puebla, MEX; 8 Medicine, Facultad de Medicina Universidad Autónoma de San Luis Potosí, San Luis Potosi, MEX; 9 Division of Research & Academic Affairs, Larkin Community Hospital, South Miami, USA; 10 Internal Medicine, Larkin Community Hospital, South Miami, USA

**Keywords:** narrative review, diagnosis, treatment, genetic, low income, high income, left ventricular hypertrophy, hypertrophic cardiomyopathy

## Abstract

Hypertrophic cardiomyopathy (HCM) is a hereditary cardiac condition characterized by unexplained left ventricular hypertrophy without a hemodynamic cause. This condition is prevalent in the United States, resulting in various clinical manifestations, including diastolic dysfunction, left ventricular outflow obstruction, cardiac ischemia, and atrial fibrillation. HCM is associated with several genetic mutations, with sarcomeric mutations being the most common and contributing to a more complex disease course. Early diagnosis of HCM is essential for effective management, as late diagnosis often requires invasive treatments and creates a substantial financial burden. Disparities in HCM diagnosis and treatment exist between high-income and low-income countries. High-income countries have more resources to investigate and implement advanced diagnostic and treatment modalities. In contrast, low-income countries face challenges in accessing diagnostic equipment, trained personnel, and affordable medications, leading to a lower quality of life and life expectancy for affected individuals. Diagnostic tools for HCM include imaging studies such as 2D echocardiography, cardiovascular magnetic resonance (CMR), and electrocardiograms (ECGs). CMR is considered the gold standard but remains inaccessible to a significant portion of the world's population, especially in low-income countries. Genetics plays a crucial role in HCM, with numerous mutations identified in various genes. Genetic counseling is essential but often limited in low-income countries due to resource constraints. Disparities in healthcare access and adherence to treatment recommendations exist between high-income and low-income countries, leading to differences in patient outcomes. Addressing these disparities is essential to improve the overall management of HCM on a global scale. In conclusion, this review highlights the complex nature of HCM, emphasizing the importance of early diagnosis, genetic counseling, and access to appropriate diagnostic and therapeutic interventions. Addressing healthcare disparities is crucial to ensure that all individuals with HCM receive timely and effective care, regardless of their geographic location or socioeconomic status.

## Introduction and background

Hypertrophic cardiomyopathy (HCM) is the primary hereditary cardiomyopathy that can develop at any age. It is an autosomal dominant disease characterized by unexplained left ventricular hypertrophy without a hemodynamic cause ​[1-4]. In the United States, the prevalence of hypertrophic cardiomyopathy is 1 in 500 adults, or around 0.2%, with a pediatric incidence of 0.24-0.47 per 100,000 per year ​[1,5-9]. HCM presents with equal distribution by sex [3,10]. It has numerous etiologies, including sarcomeric disorders, inborn metabolic errors, RASopathies, mitochondrial myopathies, and glycogen and lysosomal storage disorders in children ​[2,3,11]. Several genes have been implicated in the pathogenesis of HCM, including myosin-binding protein C3 (MYBPC3) and beta myosin heavy chain 7 (MYH7) gene mutations, that correspond to sarcomeric (thick filament protein) and thin filament protein-encoding genes such as TNNT2 and TNNI3 [2,3,12]. Most studies describe the predominance of sarcomeric mutations in a more complex disease course, with left ventricular hypertrophy (LVH), myocardial fibrosis, arrhythmias, and heart failure. HCM patients with sarcomeric mutations have a disease onset at around 40 years [7,13,14]. Additionally, oxidative stress has been described as a primary element in developing cardiac muscle hypertrophy and fibrosis [13,14].​ Disease progression and cardiac dysfunction are expected clinical outcomes, with diastolic dysfunction, left ventricular (LV) outflow obstruction, cardiac ischemia, and atrial fibrillation being the most common ​[9].​ A few studies also consider sudden cardiac death as one of the most common outcomes in young patients with sarcomeric mutations [7,8,13,14]. Patients with left ventricular outflow obstruction account for approximately two-thirds of HCM patients. On the other hand, atrial fibrillation results from cardiac fibrosis in older patients [12]. Risk score parameters and cardiovascular magnetic resonance imaging of the LV may be helpful in fatal arrhythmia prevention [9]. The term “idiopathic hypertrophic sub-aortic stenosis” has historically been used to describe HCM [10,15]. However, diagnostic difficulties may coexist phenotypically with HCM, so misdiagnosis is common. Clinical manifestations include dyspnea on exertion, orthopnea, chest pain, palpitations, syncope, fatigue, and edema [4,6]. Clinical diagnosis of hypertrophic cardiomyopathy can be made by imaging studies, such as 2D echocardiography or cardiovascular magnetic resonance (CMR) [4]. Patient evaluation should begin with a detailed family history going back at least three generations, a comprehensive physical examination of the patient, followed by an electrocardiogram and cardiac imaging specific to the left ventricle is needed [3,6]. The echocardiogram is considered the gold standard of diagnostic tests. Therefore, cardiac imaging is a fundamental part of the diagnosis and clinical management of HCM. Unfortunately, survival is hampered by etiology and age. Therefore, it is recommended to undergo screening for hypertrophic cardiomyopathy during puberty [1].

HCM can easily be misdiagnosed due to a lack of diagnostic equipment and overlapping symptomology. The health inequality burden not only falls upon each country, but it also affects the global community. The inability to effectively diagnose and treat diseases significantly at the later stages lowers a community’s quality of life and life expectancy. A late diagnosis of HCM requires critical and invasive treatment, which in turn creates a financial burden [16]. Early detection and treatment greatly benefit not only the patient, but society as a whole. This review aims to summarize the evidence in the disparity on the diagnosis and treatment of HCM.

## Review

Methods

This review synthesizes existing research on HCM and the differences in high- and low-income countries. Literature from various sources (PubMed, Scopus, Medline, and Google Scholar) were systematically searched and reviewed. Studies included in this review encompassed a diverse cohort of patients with HCM, covering different age groups and countries. The selection criteria included studies with patients with HCM as a diagnosis and patients from variable income countries.

High-income countries

High-income countries have the opportunity to investigate new diagnostic and treatment modalities, to improve both. Therefore, we divide the countries according to their income and the World Health Organization definition that a high-income country has more than $12,745 per capita [17].

Low-income countries

**Figure 1:**
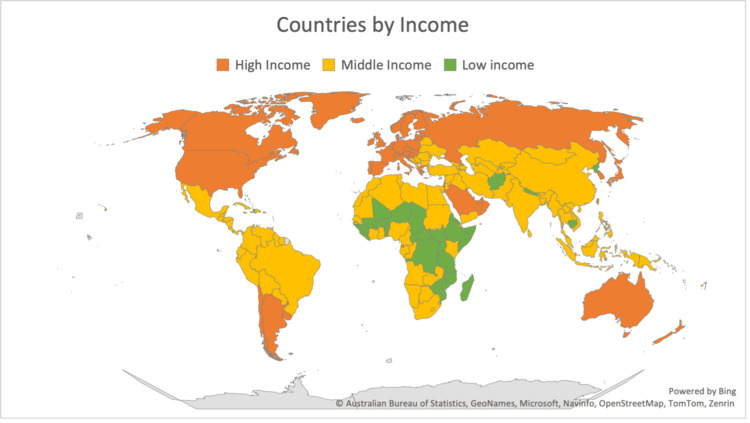
World map comparison of countries by income Based on data from the World Health Organization and Our World in Data for 2021 [17].

According to the definition by the World Health Organization, we also divide the low-income countries that have less than $1045 per capita​ [17]. This section will discuss studies performed in Nigeria, India, and other countries (Figure 1).

General considerations

To gain a clear understanding of the diagnosis, it's crucial to take into account the recent demographic changes that this pathology has experienced, as numerous parameters have been evolving. For example, a study shows that the average ventricular septal thickness has decreased markedly from 20 mm to 17 mm in older people ​[17].

Furthermore, certain studies have found that women tend to be older and experience more symptoms compared to men when meeting the criteria for Left Ventricular Hypertrophy (LVH) on an ECG. Additionally, men more commonly exhibit abnormal Q waves. Moreover, women have a higher prevalence of obstructive physiology and systolic anterior motion. In terms of left ventricular characteristics, women typically have a smaller left ventricular end-systolic volume index and a higher left ventricular ejection fraction, as indicated by studies [6,18]. There is a need to compare the cost and benefits of different studies to reach new goals for low-economic status populations (Table 1) [19-22].

**Table 1:** Comparison of price per study. Prices per study in USD [19-22]​

Study	Diagnosis or Follow-Up	Assessment	Average Outpatient National Cost, USA	Medicaid
MRI	X		1325 USD	100 USD
X-Ray		X	125 USD	18 USD
Echography	X	X	566 USD	144 USD
EKG	X		624 USD	38 USD

Diagnosis

Findings of left ventricular hypertrophy that can’t be explained by other pathology, myocyte disorganization, and cardiac interstitial fibrosis warrant a diagnosis of HCM. As described in previously mentioned studies, this diagnosis accounts for a common clinical outcome that current pharmacological treatments have not prevented or reversed [14].

**Figure 2:**
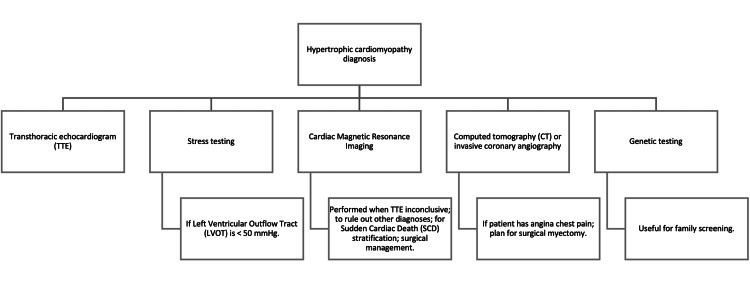
Hypertrophic cardiomyopathy diagnosis Hypertrophic cardiomyopathy diagnosis - based on 2020 AHA/ACC Guideline for Hypertrophic Cardiomyopathy: Key Perspectives [27].

In one of the studies, Regadenoson, an adenosine receptor agonist, was utilized as a vasodilator during stress myocardial contrast echocardiography to identify perfusion defects in patients with septal variant HCM. Regadenoson is available in the United States under brand name and generic. The price varies from 30 to 370 dollars depending on the generic or brand [23,24]. There is a vast amount of pharmacy discount programs available for patients in the United States and the ability to obtain the generic drug at a discounted price, in contrast to other parts of the world, like Mexico, where only brand name drug is available. The disparity in availability and consumer reach widens the gap between high- and low-income countries. Patients in low-income countries do not have the same opportunity to obtain the drug and are put at a disadvantage with possible life-threatening consequences. Many patients tested positive, resulting in septal perfusion defects and hyperemia. After septal myomectomy, hyperemia showed a marked improvement (Figure 2) ​[25]. In a study performed in Nigeria, there was a prevalence of 5.7% for hypertrophic cardiomyopathy in a cohort of 594 people referred for echocardiographic assessment. Most cases with progressive myocardial damage show an evident need for better and earlier ways to diagnose this condition in this population ​[26]. For HCM diagnosis, echocardiography and cardiovascular magnetic resonance (CMR) are established imaging strategies for clinical diagnosis [10].

Magnetic resonance

Magnetic resonance is an essential tool in the diagnosis of Hypertrophic Cardiomyopathy. It helps diagnose unknown conditions and refute suspected cardiomyopathy ​[28]. For magnetic resonance, a definitive diagnosis of HCM is made when the pattern of left ventricular hypertrophy (LVH) is asymmetrical with a maximal left ventricular (LV) wall thickness >15 mm in the absence of other causes. In subjects with a family history of HCM, a lesser degree of LVH is diagnostic, only requiring >13 mm of wall thickness. Definitive diagnosis of HCM was made in patients demonstrating non-contiguous areas of hypertrophy [29]. End Stage-HCM (ES-HCM) patients present with ventricular remodeling and extensive late gadolinium enhancement (LGE). The replacement fibrosis or interstitial fibrosis detected by LGE is one of the essential features in ES-HCM, a prognostic value tool for adverse outcomes [28].

Cardiovascular magnetic resonance is the gold standard for many cardiac pathologies and has shown to be superior to other imaging techniques [30]. Despite the essential role of MRI in diagnosis and its many advantages over other imaging modalities, it has been estimated that 66% of the world does not have access to this diagnostic tool - low-income countries are most affected [31]. The costs of acquisition and siting of MRI being upwards of $1 million added to minimal budget investment towards health care by low-income countries have made MRI less accessible [30,31].

Late gadolinium enhancement

It has been associated with the replacement or interstitial fibrosis, increased maximal septal thickness mean, and mean septal to free wall thickness ratio ​[32]. In addition, LGE is a marker that predicts a lower risk for sudden cardiac death and the development of end-stage HCM with systolic dysfunction ​[33].

Echocardiography

The current diagnostic criteria for HCM in echocardiography are an increase in left ventricular (LV) wall thickness >15 mm in at least one myocardial segment or >13 mm for patients with a first-degree relative with confirmed HCM. The measurement of the LV wall in parasternal short-axis views at end-diastole is the most accurate to evaluate these patients. In addition, asymmetric hypertrophy with a septal-to-posterior wall thickness ratio >1.3 in normotensive patients or >1.5 in hypertensive patients may also suggest HCM ​[34].

Echocardiography offers several benefits, including the ability to identify systolic anterior motion and mechanical impedance as potential causes of left ventricular outflow obstruction. It can also help assess other mechanisms of obstruction, such as muscle in the central cavity, provide quantitative estimates of peak instantaneous left ventricular outflow gradient, evaluate the extent of mitral regurgitation, and assess the condition of the aortic and mitral valves. This evaluation may reveal various abnormalities, such as elongated mitral leaflets or left ventricular diastolic dysfunction (LVDD) [34,35].

One of the limitations for low-income countries is the amount of money and time required to train a physician in the use of ultrasonography and there is a need to adapt the curriculum of the medical students into the use of ultrasonography such as point-of-care ultrasound, which has demonstrated an increase of sensitivity and specificity in disease detection in several settings [36-38].

Strain

The circumferential transmural strain difference (cTSD) is the difference between an epicardial and endocardial circumferential strain. It is a valuable tool to evaluate HCM in patients with sarcomere mutations and even in carriers of preclinical sarcomere mutations. These mutations result in early or occult functional abnormalities such as increased force generation [39,40].

Electrocardiogram

An electrocardiogram (ECG) is a valuable tool for providing information about potential diagnoses, prognoses, and follow-up in medical practice. Late gadolinium enhancement in imaging has been linked to increased septal thickness and an unusual decrease in precordial voltages on ECG, specifically lower voltages in SV1 and SV1+RV6, along with an increase in septal dimensions. It's important to note that while ECG can provide useful information, it cannot replace the need for magnetic resonance imaging (MRI). However, it can help reduce the frequency of MRI scans or indicate the need for earlier testing [32].

In addition to ECG interpretation, it's crucial to invest in personnel training and retention to manage the costs associated with diagnosis, prognosis, and follow-up. To this end, an algorithm has been developed that shows a similar diagnostic rate, sensitivity, and specificity as expert physicians in diagnosing hypertrophic cardiomyopathy (HCM) based on ECG parameters. These parameters include a Q wave depth of 0.275 mV, Q wave duration of 80 ms, ST depression of 0.008 mV, and T wave inversion of 0.12 mV. For optimized specificity, a cutoff of >3.9 mV is used for SV1+RV6, and axis deviation is considered between -30 and -90 degrees. This optimization is crucial in reducing false positives and the need for additional diagnostic tests like MRI, thereby minimizing associated costs [41-43].

Electrocardiogram (ECG) is the most common method for screening and diagnosing cardiovascular disease [44,45]. However, rural communities of low-income countries do not have access to this diagnostic tool due to their unreliable electricity supply, high cost and limited access to healthcare workers trained to read an ECG [44,45]. In remote communities of developing countries, there is often no electricity at all, and sometimes their residents may travel long ways to get access to a healthcare unit. For this reason, there have been projects aimed to develop devices of low cost and devices that do not need any electricity [44,45]. For example, in 2016, a project led by US physicians worked to create a device that reduces the cost of ECG from approximately $2,000-10,000 to $350, allowing for the screening of patients with cardiovascular diseases (CVD) without the need for a physician to be present in rural communities. This device sends information to an off-site doctor [45].

Genetics

From the perspective of the genetic field, HCM is the most common inherited cardiovascular disease that affects approximately 1 in 500 people.

Over 1500 mutations in 26 different genes have been identified as the causes of this disease. These genes include nine sarcomeric genes and nine non-sarcomere-encoding genes. Among these mutations, those involving myosin-binding protein C (MYBPC3) and Beta myosin heavy chain (MYH7) are the most common, contributing to more than half of hypertrophic cardiomyopathy (HCM) cases. This prevalence may be attributed to the substantial size of these genes, both exceeding 3000 base pairs.

Interestingly, when it comes to echocardiographic findings, there are no significant differences between patients with mutations in these two genes. However, carriers of these mutations exhibit a higher left ventricular ejection fraction and a notable degree of variability in phenotypic expression and disease penetrance.

Furthermore, there are differences in symptomatology among patients with mutations in these genes. Patients with MYBPC3 mutations tend to experience dyspnea more frequently, while those with MYH7 mutations report palpitations and a higher incidence of atrial fibrillation. Additionally, calcification of the mitral annulus is observed more frequently in patients with MYH7 gene mutations, and these individuals tend to exhibit more pronounced disease severity [46-48].

It has been reported that patients with thin filament mutations showed less prevalent outflow tract obstruction and a higher rate of progression to New York Heart Association Functional class III or IV, higher prevalence of systolic dysfunction, or restrictive LV filling [22]. Although patients with sarcoma mutations, whether thin or heavy filament, were more likely to have reverse septal curvature morphology, and those sarcomere negatives were more likely to have isolated basal septal hypertrophy, less LGE and more left ventricular outflow tract (LVOT) obstruction [49].

Genetic counseling is an essential part of HCM approach. Its objective is to inform patients and families about the genetic aspects of their disease and the possibility of transmitting the disease to their relatives [50]. However, accessibility to genetic testing is limited across low-income countries; this can be due to limitations in human, technical, and financial resources [50,51].

Therapeutics

Treatment for HCM aims to manage symptoms and prevent complications such as heart failure, arrhythmias, and sudden cardiac death ​[52]. This article will explore the therapeutic options available for patients with HCM.

Special centers

The creation of dedicated centers of cardiology is one approach to the diagnosis and treatment of HCM. Nevertheless, previous knowledge demonstrated that the implementation of dedicated centers for this kind of disease will produce better management in the population that generally does not focus on this kind of disease and priority for many other cardiac conditions such as coronary artery disease or systemic hypertension [53,54]. Some studies demonstrated that patients with lower socioeconomic status have lower rates of access to these centers than those with higher socioeconomic status [55,56]. Also, a study reports a cost increase over two years after the HCM diagnosis due to hospitalizations and surgical costs ​[57].

Medications

Medications are commonly used to manage symptoms and prevent complications in patients with HCM. They do not modify disease progression [12,13]. Beta-blockers and calcium channel blockers are the mainstays of medical therapy, as they reduce the heart's workload and improve heart muscle relaxation. These medications also help prevent arrhythmias, which can be life-threatening in patients with HCM ​[58]. Anticoagulants may also be prescribed to prevent blood clots from forming in the heart, which can lead to stroke. In some cases, medications such as disopyramide may be used to reduce the obstruction caused by the thickened heart muscle ​[59,60].​

Multiple clinical trials have tested medications and molecules to prevent disease progression and reduce the economic burden of the disease for the countries. Some medications that will be discussed in this text are ranolazine, valsartan, N-acetylcysteine, spironolactone, and mavacamten (Table 2).

**Table 2:** Drugs in HCM progression HCM: Hypertrophic cardiomyopathy

Authors	Drug	Outcome
Camici et al. [12]	Ranolazine	No benefit as an intervention, and functional capacity was not improved. No impact in Brain Natriuretic Peptide (BNP) decrease, quality of life and exercise performance improvement.
Ho et al. [13]	Valsartan	Studies suggest that patients with sarcomeric mutations (MYH7 and MYBPC3) can benefit from its use early in the disease. The described rationale accounts for inhibiting transforming growth factor-beta (TGF-b), a cytokine involved in myocardial hypertrophy and fibrosis.
Marian et al. [14]	N-acetylcystein	No significant effects were confirmed.
Maroon et al. [58]	Spironolactone	Spironolactone showed promising results in murine models. However, human clinical trials did not show a decrease in collagen degradation and adequate collagen synthesis as expected, leaving space for other investigational products.
Xie et al. [16]	Mavacemten	Demonstrated a significant impact, and patients' scores improved; patients improved at least one NYHA functional class.

Surgical interventions

In severe cases of HCM, surgical interventions may be necessary to manage symptoms and prevent complications. For example, septal myectomy is a surgical procedure in which part of the thickened heart muscle is removed to improve blood flow through the heart. This procedure is a gold standard for septal reduction and evidence has shown that surgical myectomy reverses heart failure symptoms by abolishing obstruction, restoring normal LV filling pressure, and reducing mitral regurgitation [61].

Another surgical option is alcohol septal ablation, which involves injecting alcohol into a small artery in the heart to destroy a portion of the thickened heart muscle selectively. This procedure is less invasive than septal myectomy and can improve symptoms ​[62].

Sometimes, an implantable cardioverter-defibrillator (ICD) may be recommended to prevent sudden cardiac death. This device is implanted under the skin and delivers a shock to the heart if it detects a life-threatening arrhythmia. ICDs effectively reduce the risk of sudden cardiac death in patients with HCM who are at high risk for this complication [63,64].

Although surgical procedures have shown to reverse symptoms of HCM and promote long-term survival, approximately 93% of people from low-income countries lack access to cardiac surgical care [65,66]. Recent data reported that high-income countries possess approximately 7.15 adult cardiac surgeons per million population in contrast to low-income countries, which possess 0.04 adult cardiac surgeons per million population [66].

General interventions

In addition to diagnosis, medical and surgical therapies, lifestyle modifications are essential for managing symptoms and preventing complications in patients with HCM (Table 3). Patients are advised to avoid strenuous exercise and competitive sports, which can trigger symptoms and increase the risk of complications. Maintaining a healthy weight, managing stress, and avoiding alcohol and drugs are essential for managing symptoms and preventing complications. Regular cardiac monitoring, including electrocardiograms (ECGs) and echocardiograms, is recommended to detect changes in heart function ​[63]. As expected, patients in high-income countries tend to better adhere to these recommendations. These patients have more readily access to regular cardiac monitoring and the ability to improve their diet. On the other hand, patients with CVD from low-income countries have less adherence to pharmacologic treatment [67]. Strong evidence suggests that socioeconomic factors and healthcare access are significant contributors to medication non-adherence in these patients, including long distances from treatment settings, high cost of medicines and limited drug supply, family size, local beliefs about the origin of illnesses, and concerns about medical cost [67]. Lower socioeconomic groups have limited access to the clinic setting and management by a trained cardiac physician. Those patients from lower socioeconomic groups have worse health-related quality of life, lower understanding of HCM, and more complications due to poor adherence to treatment and recommendations [67].

**Table 3:** Hypertrophic cardiomyopathy low-income/high-income country differences in diagnosis and treatment

Diagnosis studies		
Study	Low-income country	High-income country
Magnetic Resonance	Low-income countries struggle with lower income per capita and less Magnetic Resonance Imaging (MRI) units per million population, for example, Mexico, Ghana, Sub-Saharan Africa, Colombia, and India have 2.9, 0.5, 0.31, 0.24, and 0.21 respectively MRI units per million population [31]. A cross-sectional study in 2018 was performed in Peru by Moon et al. who developed and tested a 15-minute CMR protocol which cost $150 USD, and the average scan duration was 18 minutes. Findings impacted management in 56% of patients, including previously unsuspected diagnoses in 19% and therapeutic management changes in 37% of patients [68]. The INCA-Peru rapid CMR protocol was modified and adapted by Menacho et al. In 2022, it was estimated cost savings for rapid CMR compared with conventional contrast CMR were between 30 and 60% cheaper, with a range of 25–30% in Cape Town, 30% in Argentina, 40% in Cuba, and 50–60% in Peru [30].	High-income countries have more Magnetic Resonance Imaging (MRI) units per million population, for example, Japan, USA, Korea, Norway, Canada, and Poland have 55.21, 34.54, 32.03, 19, 10.06, and 9.28 respectively MRI units per million population [31]. Murphy et al. in 2021 performed a cost analysis at a hospital in London, UK. It was estimated the cost for a CMR is £385 [69]. In the USA cost ranges are between $380 USD to $5,000 USD depending on the hospital and if it's covered by the insurance [69]. Śpiewak et al. in 2021 Poland did a retrospective study and evaluated the impact of CMR over a period of 10 years, and a total of 1006 patients were evaluated. CMR led to clear HCM diagnosis in 44.7% of patients, some of them previously evaluated with echocardiography. In patients with a history of uncontrolled hypertension and suspected of having HCM, CMR identified cardiomyopathy in 47.9% of patients [29].
Late gadolinium enhancement Cardiac Magnetic Resonance	Gangadharakaimal et al. in 2023 performed a study in 80 patients in India. LGE was present in 43.9% of patients. They found that an LGE in 4 or more segments was associated with ventricular arrhythmias [70].	Rubinshtein et al. in 2010 performed a study in 424 patients in Mayo Clinic. Among the 424 patients, 56% had LGE on CMR, ranging from 0.4% to 65% of the left ventricle. LGE was not associated with severe symptoms. However, LGE was strongly associated with surrogates of arrhythmia and remained a significant associate of subsequent SCD and/or ICD discharge after controlling for other variables [71].
Echocardiography	Echocardiography is an operator-dependent study which implicates the need for special equipment and personnel that can perform the echocardiogram; most studies performed in low-income countries don't include big populations. Oyedeji et al. in 2014 performed a study in Nigeria with 168 patients, the echocardiogram was normal in 64.3% of the subjects and found hypertrophic cardiomyopathy in 9% of the patients [72]. In a country where there are limited diagnostic facilities, echocardiograms could be used for the classification of HCM and for monitoring patients [73].	High-income countries usually are the first ones to provide newer diagnosis methods to patients, that is why clinical trials are usually performed in these countries and are the ones that provide strong evidence. Echocardiography has become essential for establishing the diagnosis, evaluating the extent of disease, and risk stratification [74].
Electrocardiogram	To date, the ECG remains an irreplaceable first step when evaluating patients with hypertrophic cardiomyopathy (HCM), and an abnormal ECG may be the only manifestation of the disease at an early stage [75]. Even though it is a cheap diagnosis test to run, low-income countries still need more electrocardiogram units to address protocols in patients with hypertrophic cardiomyopathy, that is why efforts like Walker et al. and Ertola et al. to create low-cost electrocardiogram bring hope to patients in low-income countries [44,45].	In high-income countries, electrocardiogram units are available even in primary care units. Despite the recent exponential development of imaging techniques and molecular diagnosis methods, the interpretation of the electrocardiogram provides information in the early identification of hypertrophic cardiomyopathy [75].
Genetics	Even though hypertrophic cardiomyopathy is caused by mutations in genes encoding proteins of the sarcomere protein in 50–70% of the cases, genetic tests are usually limited in diagnosis methods in low-income countries [76]. Zhong et al. described barriers for genetic services in Kenya in 2021: limited genetic knowledge, lack of practice guidelines and equipment, high cost, lack of insurance coverage, and laboratory capacity limitations [51].	Genomic medicine has been developed in high-income countries, though in decades the material and equipment have become more sensible and specific to detect genomic alterations. Studies that have the most impact on the quantity of patients have been performed in these countries. The continuous research allows to discover new genomic illnesses and set basis to new therapies [77].
Therapy options		
Therapy	Low-income country	High-income country
Medication	A systematic review performed by Ogungbe et al. in 2021 described patients' medical treatment adherence. Interventions that were more effective at improving medication adherence included changing from multi-dose medications to fixed-dose combinations, and team-based healthcare [67]. Usually, drugs that have been used since 1960 for treating hypertrophic cardiomyopathy are beta-blockers and calcium channel blockers, they are cheap medications and accessible to the population [78,79]. New molecules that have been discovered recently and the high cost of them, create an access barrier. Latado et al. in 2021 analyzed the cost-effectiveness relation of Evolocumab in Brazil and concluded that might not have the best relation specially with the cost [80].	Other drugs that have been used in the medical treatment of hypertrophic cardiomyopathy and that are still being studied are Disopyramide, Cibenzoline, Ranolazine and Eleclazine, Sacubitril/Valsartan, Perhexiline, and Trimetazidine [81]. Mavacamten is a novel specific myosin inhibitor that has recently been identified through a chemical screening for molecules decreasing the ATPase rate of myosin in bovine myofibrils, this new molecule seems to be promising in the prognosis of the patients [82].
Surgical interventions	Even though septal myectomy has been the gold standard for septal reduction, the procedure requires specialized centers, equipment, and personnel, that might be hard to access in low-income countries [83]. In 1999, it was estimated that there were only 4000 cardiac centers worldwide that performed cardiac surgery. Less than 1 center per 10 million population was located in low-income countries. Asian countries had only 1 per 16 million population and the African continent had 1 per 33 million population [66].	High-income countries are estimated to have more than 100 times as many cardiac surgeons per million population. In 1999, it was estimated that approximately 4000 centers performed cardiac surgery around the world, where North America possessed 1 center per 120,000 population and Europe and Australia reportedly had 1 center per 1 million population [66].

## Conclusions

Cardiovascular disease is a significant contributor to noncommunicable diseases (NCDs) in developing nations, with around 75% of NCD-related deaths occurring in middle- and low-income countries, with half of these attributed to cardiovascular diseases (CVD). Hypertrophic cardiomyopathy (HCM), once a challenging heart condition, has become more treatable thanks to advanced management options such as implantable cardioverter defibrillators, heart transplants, surgical myectomy, and alcohol septal ablation. Additionally, high-resolution imaging techniques like MRI play a crucial role in diagnosing HCM promptly and accurately.

However, limited access to these treatments in low-income countries has led to a rise in non-communicable diseases related to cardiovascular issues. This disparity is fueled by the substantial demands for training, capital investment, and specialty medical services needed for cardiovascular care. High-income countries, in contrast, have significantly more cardiac surgeons and allocate a larger share of their GDP to healthcare. Consequently, despite advances in diagnosis and treatment, HCM remains a common genetic heart disease causing substantial morbidity and mortality in low-income countries. Bridging the gap in access to modern diagnostics and management strategies holds the potential to improve outcomes and the quality of life for HCM patients in these regions.
